# Sex differences in the rate and factors for elevated depressive symptoms in multiple sclerosis, inflammatory bowel disease, and rheumatoid arthritis

**DOI:** 10.1177/13591053251396478

**Published:** 2026-01-16

**Authors:** Hayley Riel, Francisca Lois Jacobo, Charles N. Bernstein, James M. Bolton, John D. Fisk, Lesley A. Graff, Carol Hitchon, Chantel Mayo, Scott B. Patten, Ruth Ann Marrie, Kaarina Kowalec, CIHR Team in Defining the Burden and Managing the Effects of Psychiatric Comorbidity in Chronic Immunoinflammatory Disease

**Affiliations:** 1 University of Manitoba, Winnipeg, MB, Canada; 2 Dalhousie University, Halifax, NS, Canada; 3 University of Calgary, AB, Canada; 4 Nova Scotia Health, Halifax, NS, Canada; 5 Karolinska Institutet, Solna, Sweden

**Keywords:** depressive symptoms, sex, multiple sclerosis, rheumatoid arthritis, inflammatory bowel disease

## Abstract

In immune-mediated inflammatory diseases (IMID), females report elevated depressive symptoms more frequently than males. We examined clinical and sociodemographic factors associated with elevated depressive symptoms in IMID and whether endorsement of individual depressive symptoms differed by sex. This study included 652 individuals with an IMID from Manitoba, Canada. Depressive symptoms were measured using the PHQ-9 and HADS-D, with IMID-specific cut-offs to identify elevated depression. Elevated depressive symptoms were present in 234 participants (36%). Females did not show higher odds of elevated depression than males. Males were more likely to endorse the HADS-D item on cheerfulness. Smoking and anxiety symptoms, but not sex, were associated with elevated depressive symptoms. These findings may help identify high-risk individuals with an IMID and comorbid elevated depressive symptoms and guide intervention.

## Introduction

Immune-mediated inflammatory diseases (IMIDs) constitute a large group of conditions marked by a dysregulated immune response and chronic inflammation. The symptoms of IMIDs are diverse but often include persistent fatigue and pain and is associated with a high rate of comorbid mental health issues (Marrie et al., 2019). IMIDs such as multiple sclerosis (MS), inflammatory bowel disease (IBD), and rheumatoid arthritis (RA) have genetic overlap (David et al., 2018), are more prevalent in females than males (El-Gabalawy et al., 2010), and share susceptibility to certain environmental factors (Monteleone et al., 2023). The estimated prevalence of IMIDs in Western countries ranges from 3% to 7%, though estimates vary depending on diagnostic criteria and the population studied (El-Gabalawy et al., 2010; Monteleone et al., 2023). Given the chronic and burdensome nature of these diseases, individuals with IMIDs are at elevated risk for mental health comorbidities, particularly depression.

Elevated depressive symptoms are 2–4x higher in the IMID population compared to the general population (Graff et al., 2009; Marrie et al., 2018a; Moulton et al., 2019; Uda et al., 2021). Elevated depressive symptoms can be sufficient to constitute a diagnosis of major depressive disorder (MDD); however, if they do not meet diagnostic criteria these symptoms still adversely affect individuals with an IMID and may increase the risk for a future MDD diagnosis (Bhandari et al., 2017; Feinstein et al., 2014; Isik et al., 2007; Matcham et al., 2013; Skokou et al., 2012). In the general population, females demonstrate greater severity and frequency of depressive symptoms than males (Angst and Dobler-Mikola, 1984; Marcus et al., 2005, 2008). Based on depressive symptom scales, females report a higher increase in appetite, weight problems, gastrointestinal symptoms, and more interpersonal sensitivity compared to males (Marcus et al., 2005). Given that sex differences in susceptibility to IMIDs are noticeable (Al-Sakran et al., 2018; Bernstein et al., 2006; El-Gabalawy et al., 2010; Hitchon et al., 2020a; Jacobson et al., 1997), as are differences in disease severity (Iikuni et al., 2009; Lesuis et al., 2012; Sokka et al., 2009; Wagtmans et al., 2001), the association of sex and depressive symptoms may also differ in IMIDs and general populations. Recent evidence continues to support the high psychiatric burden in individuals with IMIDs and the persistence of sex differences in symptom presentation (Joyees et al., 2023; Kowalec et al., 2022; Marrie et al., 2019). The limited studies that have explored sex differences in depressive symptoms in IMID have mostly focused on MS (Mayo et al., 2021; Solaro et al., 2016; Théaudin et al., 2016), despite frequent depressive symptoms in RA and IBD as well (Bhandari et al., 2017; Matcham et al., 2013; Moulton et al., 2019; Whitehouse et al., 2019). One prior study found that females displayed greater symptoms of depression than males (Mayo et al., 2021), while the others found no sex differences in depressive symptoms (Solaro et al., 2016; Théaudin et al., 2016). It is also unknown whether the characteristics of elevated depression, as identified by specific items on depressive symptom scales, differ by sex in IMIDs. Identifying factors associated with depressive symptoms in those with an IMID will help with earlier recognition, tailored treatment options, reduced mortality, and may also highlight potential biological differences in depressive symptoms by sex.

Several sociodemographic and clinical factors have been shown to influence depressive symptoms in individuals with IMIDs. Lower income, lower educational attainment, and smoking have been consistently associated with increased risk of depression in both general and IMID-specific populations (Johnston et al., 2004; Matcham et al., 2013; Schlax et al., 2019). Obesity has also been linked to depression in RA and IBD, possibly through inflammatory and psychosocial mechanisms (Bhandari et al., 2017; Moulton et al., 2019). Anxiety symptoms often co-occur with depression in chronic illness populations, particularly those with IMIDs (Gorman, 1996; Marrie et al., 2017). Illness duration and IMID type may reflect differing experiences of disease burden, symptom severity, or treatment effects that influence depressive symptoms.

The early recognition and prevention of illness in individuals with an IMID is critical in improving outcomes and reducing mortality rate (Russell et al., 2011). This cross-sectional study aimed to determine which clinical and sociodemographic factors were associated with elevated depressive symptoms in IMID, and whether they differ by sex. We also aimed to investigate the endorsement of individual items on the depressive symptom scales by sex. We hypothesized that the prevalence of elevated depressive symptoms in individuals with IMIDs is higher in females than males, and that factors such as age, income, education, smoking status, IMID type (MS, IBD, RA), illness duration, anxiety, and body mass index (BMI) were associated with elevated depressive symptoms and that their association differed by sex. We also hypothesized that the endorsement of individual items on the depressive symptom scales would differ by sex.

## Methods

### Study design and participants

This analysis implemented a cross-sectional study design to investigate sex differences in depressive symptoms. We used participants from a pre-existing cohort investigation of psychiatric comorbidities in IMID (Marrie et al., 2018b). Participants were from Manitoba, Canada and were recruited between November 2014 and July 2016 (study protocol published in Marrie et al., 2018b). Participants were recruited in-person or by mail outreach to patients from community-based and tertiary clinics and through poster placements in hospitals, private medical clinics, educational institutions, social media platforms such as Twitter and Facebook, and mental health support groups. Inclusion criteria: at least 18 years of age, capable of providing informed consent, and sufficiently proficient in English to complete the questionnaires. In addition, all participants had a confirmed diagnosis of MS, RA, or IBD using standard diagnostic criteria for the respective disease (Aletaha et al., 2010; Bernstein, 1997; Marrie et al., 2018b; Silverberg et al., 2005). No exclusion criteria applied. This study was conducted in accordance with the Declaration of Helsinki (2013), and the Fortaleza Amendment (2013 WMA General Assembly).

### Factors for elevated depressive symptoms

At baseline, participants were given a self-report questionnaire that asked about sex where the options were “male” or “female.” The questionnaires also collected sociodemographic and clinical information such as annual household income (in Canadian dollars: ⩽ $50,000, ⩾ $50,000, or “Declined to answer”), educational attainment (high school or below, above high school), and smoking history (participants who reported having ⩾100 cigarettes during their lifetime were categorized as ever smokers, while the rest were categorized as none-to-moderate smokers). Height and weight were measured by research assistants to calculate BMI (kg/m^2^). Illness duration was calculated as the number of years between the self-reported year of IMID onset and the baseline study visit.

### Psychiatric symptoms

Elevated depressive symptoms were identified at baseline using two scales: the Patient Health Questionnaire (PHQ-9; Spitzer et al., 1999) and the Hospital Anxiety and Depression Scale (HADS; Zigmond and Snaith, 1983). These scales have been validated in all three IMID populations (Bernstein et al., 2018; Hitchon et al., 2020b; Marrie et al., 2018c), with high internal consistency as measured with Cronbach’s alpha of ⩾0.70 (Bland and Altman, 1997): PHQ-9 in IBD (0.89, 95% CI [0.82, 0.95]), RA (0.89, 95% CI [0.80, 0.97]), MS (0.87, 95% CI [0.80, 0.94]) and HADS-D in IBD (0.84, 95% CI [0.76, 0.92]), RA (0.84, 95% CI [0.74, 0.94]), and MS (0.82 95% CI [0.75, 0.90]) (Bernstein et al., 2006; Hitchon et al., 2020a; Marrie et al., 2018b). Incorporating both scales enhanced sensitivity of identifying elevated depressive symptoms. The PHQ-9 is a self-report questionnaire used to assess the severity of depressive symptoms experienced over the preceding two weeks through nine questions, each employing a four-point scale from 0 (not at all) to 3 (nearly every day). A PHQ-9 score ⩾ 10 signifies the presence of current elevated depressive symptoms in the general population(Kroenke et al., 2001), and a score of PHQ-9 ⩾ 10 (RA), PHQ-9 ⩾ 11 (IBD), and PHQ-9 ⩾ 12 (MS) indicates elevated depressive symptoms in the respective IMIDs (Bernstein et al., 2018; Hitchon et al., 2020b; Marrie et al., 2018c). The HADS has two subscales, one for depressive symptoms (HADS-D) and the other for anxiety symptoms (HADS-A). Each subscale evaluates depression or anxiety symptoms experienced within the previous week of completing the questionnaire and is comprised of seven questions utilizing the same four-point scale, yielding a total range of 0 to 21. A HADS-D and HADS-A score of ⩾11 indicates probable depressive and anxiety symptoms in the general population, and cut-points of ⩾7 and ⩾6 for probable depressive and anxiety symptoms, respectively, in specifically MS, IBD, and RA (Bernstein et al., 2018; Hitchon et al., 2020b; Marrie et al., 2018b). As such, IMID participants were categorized as exhibiting current elevated depressive symptoms if they had a HADS-D ⩾ 7 (any IMID) or PHQ-9 ⩾ 10 (RA), PHQ-9 ⩾ 11 (IBD), and PHQ-9 ⩾ 12 (MS). In addition, to explore whether specific depressive symptoms differed by sex in their manifestation, the endorsement of individual items on the PHQ-9 or HADS-D were categorized as ⩾1 versus no symptoms.

### Physical symptoms

Pain and fatigue are common symptoms of IMIDs, so in addition to elevated depressive symptoms, pain was measured using the Modified Pain Effects Scale (MPES; Ritvo et al., 1997a, 1997b) and fatigue was measured with the Fatigue Impact Scale for Daily Use (DFIS; Fisk and Doble, 2002). The MPES is a six-item questionnaire with total scores ranging from 6 to 30, providing an assessment of the impact of pain on mood, quality of life, ability to move, sleep, work, and execute recreational activities. The DFIS is a validated 8-item measure of the experience with daily fatigue; items are scored from 0 (no) to 4 (extreme problem). On the MPES and DFIS, higher scores indicate greater impact of pain and fatigue, respectively.

### Data analyses

Participant characteristics, including DFIS and MPES scores, were described overall and by sex using the mean (standard deviation) or frequency (%), as appropriate in Table 1. Characteristics between males and females with an IMID who met the threshold for elevated depressive symptoms were compared to those who did not meet this threshold using a *t*-test for continuous variables or chi-square test for categorical variables.

**Table 1.** IMID types, sociodemographic, and factor information of the IMID cohort.

Factor	Total IMID cohort	Elevated depressive symptoms	No elevated depressive symptoms	(1) Males with elevated depressive symptoms	(2) Females with elevated depressive symptoms	(1) vs. (2) *p*-value
	N (%)					
Participants	652 (100)	234 (35.89)	418 (64.11)	51 (21.79)	183 (78.21)	<0.001*
Sex (female)	492 (75.46)	183 (78.21)	309 (73.92)	-	-	-
*IMID type*						
MS	254 (38.96)	95 (40.60)	159 (38.04)	19 (37.25)	76 (41.53)	<0.001*
IBD	245 (37.58)	78 (33.33)	167 (39.95)	25 (49.02)	53 (28.96)	0.002*
IBD: Crohn’s disease	152 (62.04)	55 (70.51)	97 (58.08)	15 (60.00)	40 (75.47)	0.001*
IBD: Ulcerative colitis	93 (37.96)	23 (29.49)	70 (41.92)	10 (40.00)	13 (24.53)	0.53
RA	153 (23.47)	61 (25.64)	92 (22.01)	7 (13.73)	54 (29.51)	<0.001*
*Body Mass Index type*						<0.001*
Underweight-normal	229 (35.12)	70 (29.91)	159 (38.04)	12 (23.53)	58 (31.69)	
Overweight	217 (33.28)	79 (33.76)	138 (33.01)	24 (47.06)	55 (30.05)	
Obese	198 (30.37)	80 (34.19)	118 (28.23)	12 (23.53)	68 (37.16)	
*Annual household income*						<0.001*
⩾50,000	207 (31.75)	96 (41.03)	111 (26.56)	22 (43.14)	74 (40.44)	
<50,000	388 (59.51)	118 (50.43)	270 (64.59)	26 (50.98)	92 (50.27)	
Declined	57 (8.74)	20 (8.55)	37 (8.85)	3 (5.88)	17 (9.29)	
*Education*						<0.001*
⩾High school	442 (67.80)	141 (60.26)	301 (72.01)	28 (54.90)	113 (61.75)	
<High school	210 (32.21)	93 (39.74)	117 (27.99)	23 (45.10)	70 (38.25)	
*Smoking status*						
None-moderate	266 (40.80)	74 (31.62)	192 (45.93)	13 (25.49)	61 (33.33)	<0.001*
Ever smoker	386 (59.20)	160 (68.38)	226 (54.07)	38 (74.51)	122 (66.67)	
	Mean (SD)					
Age (at baseline)	51.71 (14.14)	49.93 (12.87)	52.71 (14.71)	52.70 (16.08)	50.00 (12.57)	0.88
Body Mass Index	28.29 (6.76)	29.06 (7.14)	27.87 (6.51)	27.82 (6.73)	29.21 (7.40)	0.49
Illness duration (years)	20.28 (12.08)	20.01 (11.87)	20.42 (12.21)	21.54 (12.46)	20.37 (11.80)	0.39
PHQ-9 Score	6.81 (5.78)	12.79 (4.87)	3.38 (2.60)	3.55 (2.61)	12.86 (4.74)	0.72
HADS-D Score	4.56 (3.76)	8.45 (3.05)	2.37 (1.87)	2.44 (1.89)	8.24 (3.02)	0.05
HADS-A Score	6.27 (4.12)	9.39 (3.74)	4.52 (3.20)	4.83 (3.22)	9.63 (3.78)	0.05
Modified Pain Effects Score	13.24 (5.76)	17.88 (5.05)	10.65 (4.33)	11.00 (4.36)	18.00 (4.88)	0.51
Fatigue Impact Scale for Daily Use Score	10.09 (8.14)	16.53 (7.65)	6.47 (5.86)	7.03 (6.00)	16.79 (7.62)	0.33

Body mass index (data missing for eight participants); Modified Pain Effects scale (data missing for five participants); Fatigue Impact Scale for Daily Use (data missing for one participant). Elevated depressive symptoms are defined as: HADS-D ⩾7 (any IMID) or PHQ-9 ⩾10 (RA), PHQ-9 ⩾11 (IBD), and PHQ-9⩾12 (MS).

RA: rheumatoid arthritis; MS: multiple sclerosis; IBD: inflammatory bowel disease; PHQ-9: Patient Health Questionnaire; HADS-A: Hospital Anxiety and Depression Scale-Anxiety subscale.

* *p*-Value significance level: <0.05 using a *t*-test for continuous variables or chi-square test for categorical variables.

To determine whether sex was associated with differential endorsement of specific items on the depressive symptom scales, a series of logistic regression analyses were performed for all IMID participants with each depressive symptom scale item. We used either the PHQ-9 or HADS-D specific item as the outcome, along with sex as a predictor, and the total PHQ-9 or HADS-D score as a covariate. The odds ratio (OR), 95% confidence intervals (95% CI) and *p*-values were reported. These analyses were conducted to explore whether specific depressive symptoms differed by sex in their manifestation, rather than to formally assess measurement bias.

Univariable and multivariable logistic regression models were implemented to investigate factors associated with elevated depressive symptoms (defined as meeting the IMID-specific threshold criteria on the HADS-D or PHQ-9: yes vs no), given the outcome variable was binary. Standard assumptions for logistic regression were met. The models included the following factors, selected a priori based on their connection with depressive symptoms within other IMID studies or from the broader non-IMID literature: sex, age, IMID type, and duration of illness, BMI, annual household income, educational attainment (Johnston et al., 2004; Schlax et al., 2019; von Dem Knesebeck et al., 2011), smoking status, and anxiety based on the HADS-A given that elevated anxiety symptoms often co-occur with elevated depressive symptoms (Gorman, 1996). To identify potential sex differences, we included interaction terms for sex with each factor. Regression assumptions of independence, no multicollinearity, and linearity in the logit were met. The OR, 95% CI, and *p*-values were reported.

To mitigate any potential discrepancy of how elevated depressive symptoms were defined using the IMID-specific cut-offs, sensitivity analyses were conducted by defining (i) elevated depressive symptoms using only the HADS-D, (ii) only the PHQ-9, and then with the (iii and iv) general population cut-offs for either the HADS-D and PHQ-9. The overlap between definitions used to define elevated depressive symptoms was reported.

*R* for Statistical Computing (v4.3.1) and *R*-studio (v 2023.12.1+402) were used to analyze the data. The statistical significance level was set at *p* < 0.05 and missing data were not imputed.

## Results

The analysis included 652 (492 females, 160 males) IMID participants of whom 39% had MS, 38% had IBD, and 23% had RA. The average age of participants was 52 years with an illness duration of 20 years (Table 1). Most participants were classified as ever smokers, and across all IMIDs, there were similar proportions within each BMI category (underweight-normal, overweight, and obese). Among all participants, 36% met criteria for elevated depressive symptoms, most of whom were female (79%). The prevalence of current elevated depressive symptoms was the highest in those with MS (41%), followed by IBD (33%), and RA (26%), Table 1).

Overall, those with elevated depressive symptoms were more likely to have a lower annual household income, be obese, and have ever smoked compared to those without elevated symptoms (Table 1). Among participants with elevated depressive symptoms, females were more likely than males to have lower income, more education, a history of smoking, and obesity (*p* < 0.001). Females were also more likely to have MS, Crohn’s disease, or RA (*p* < 0.05). In those with elevated depressive symptoms, MPES scores were ~7 points higher in IMID participants than in those without an IMID. Similarly, DFIS scores averaged 10 points higher. Females reported higher average MPES and DFIS scores of 18 and 16, compared to male scores of 11 and 7, respectively. The analyses of pain and fatigue scores (MPES and DFIS) were exploratory in nature. Although females had higher mean MPES and DFIS scores, these sex differences did not reach statistical significance (*p* = 0.51 and *p* = 0.33, respectively).

### Sex differences in individual depressive symptom scale items

The most highly endorsed symptoms among IMID participants were concerns with sleep, low energy and feeling slowed down (Table 2), with over 60% of female and male participants indicating relevance of these symptoms. Most items of the depression measures showed no differentiation between males and females; however, males more often endorsed the HADS-D item pertaining to cheerfulness (item 6: “I feel cheerful”) than females (OR = 0.38, 95% CI [0.22, 0.68], *p* < 0.001; Table 2). The other results were non-significant.

**Table 2.** Logistic regression of individual items on the PHQ-9 and HADS-D in the IMID cohort (male sex was used as reference).

Outcome (Item endorsement: yes/no) PHQ-9	*N* (%) Females endorsing	*N* (%) Males endorsing	Female Odds ratio	95% CI	*p*-Value
Item 1 “Little interest or pleasure in doing things”	219 (47.61)	69 (43.13)	0.65	0.37–1.12	0.12
Item 2 “Feeling down, depressed, or hopeless”	223 (45.33)	65 (40.63)	0.85	0.50–1.45	0.56
Item 3 “Trouble falling or staying asleep, or sleeping too much”	312 (63.41)	100 (62.50)	0.81	0.51–1.28	0.37
Item 4 “Feeling tired or having little energy”	381 (77.44)	112 (70.00)	1.39	0.77–2.51	0.27
Item 5 “Poor appetite or overeating”	261 (53.05)	72 (45.00)	1.22	0.76–1.98	0.41
Item 6 “Feeling bad about yourself”	209 (42.48)	62 (38.75)	0.92	0.56–1.49	0.73
Item 7 “Trouble concentrating on things, such as reading the newspaper or watching television”	192 (39.02)	53 (33.13)	1.10	0.66–1.82	0.72
Item 8 “Moving or speaking so slowly that other people could have noticed? Or the opposite – being so fidgety or restless that you have been moving around a lot more than usual”	126 (25.61)	35 (21.88)	1.06	0.62–1.81	0.84
Item 9 “Thoughts that you would be better off dead or of hurting yourself in some way”	58 (11.79)	17 (10.63)	0.95	0.46–1.95	0.87
*HADS-D*					
Item 2 “I still enjoy the things I used to enjoy”	271 (55.08)	88 (55.00)	0.61	0.35–1.05	0.07
Item 4 “I can laugh and see the funny side of things”	152 (30.89)	46 (28.75)	1.12	0.66–1.89	0.68
Item 6 “I feel cheerful”	199 (40.45)	75 (46.88)	0.38	0.22–0.68	<0.001*
Item 8 “I feel as if I am slowed down”	387 (78.66)	118 (73.75)	1.14	0.68–1.89	0.62
Item 12 “I have lost interest in my appearance”	245 (49.80)	74 (46.25)	0.87	0.49–1.53	0.63
Item 14 “I look forward with enjoyment to things”	138 (28.05)	51 (31.88)	0.72	0.45–1.17	0.19

Missing PHQ-9 and HADS-D data for three females and one male. Adjustment for total PHQ-9 total score or HADS-D total score, respectively. Elevated depressive symptoms defined as: HADS-D ⩾7 (any IMID) or PHQ-9 ⩾10 (RA), PHQ-9 ⩾11 (IBD), and PHQ-9⩾12 (MS).

PHQ-9: Patient Health Questionnaire; HADS-D: Hospital Anxiety and Depression Scale.

* Significance level *p* < 0.05.

### Factors associated with elevated depressive symptoms

In the univariable regression model, females did not have significantly higher odds for elevated depressive symptoms (OR: 1.23, 95% CI [0.85, 1.80], *p* = 0.22, Table 3). Higher HADS-A scores, younger age, lower education level, obesity, and ever smoking were associated with increased odds of elevated depressive symptoms (Table 3). In the multivariable analysis, sex remained unassociated with elevated depressive symptoms (OR: 0.79, 95% CI [0.47, 1.33], *p* = 0.34); however, a higher HADS-A score (OR: 1.47, 95% CI: [1.38, 1.57, *p* < 0.001), and ever smoking (OR: 1.64, 95% CI [1.06, 2.56], *p* = 0.03, Table 4) were associated with elevated depressive symptoms. The multivariable regression model with sex as an interaction term for each factor found no significant sex interactions.

**Table 3.** Univariable logistic regression models of predictors of elevated depressive symptoms in the IMID cohort.

Factor	Odds ratio	95% CI	*p*-Value
Sex			
Male	-	-	-
Female	1.23	0.85–1.80	0.22
Age	0.99	0.97–1.00	0.02*
Income			
Declined	-	-	-
⩾50,000	0.81	0.45–1.47	0.48
<50,000	1.60	0.88–2.98	0.13
Education			
⩾High school	-	-	-
<High school	1.70	1.21–2.38	0.002*
Body Mass Index			
Underweight-Normal	-	-	-
Overweight	1.30	0.88–1.93	0.19
Obese	1.54	1.03–2.30	0.03*
Smoking status			
None-moderate	-	-	-
Ever smoker	1.84	1.32–2.58	<0.001*
IMID type			
RA	-	-	-
MS	0.90	0.60–1.36	0.62
IBD	0.70	0.46–1.07	0.10
IMID illness duration	1.00	0.98–1.01	0.67
HADS-A Score	1.46	1.38–1.56	<0.001*

Body mass index (data missing for eight participants). Elevated depressive symptoms defined as: HADS-D ⩾ 7 (any IMID) or PHQ-9 ⩾10 (RA), PHQ-9 ⩾11 (IBD), and PHQ-9⩾12 (MS).

RA: rheumatoid arthritis; MS: multiple sclerosis; IBD: inflammatory bowel disease; HADS-ARA = Rheumatoid arthritis; MS = Multiple sclerosis; IBD = Inflammatory bowel disease; HADS-A = Hospital Anxiety and Depression Scale. Hospital Anxiety and Depression Scale.

* Significance level *p* < 0.05.

**Table 4.** Multivariable logistic regression of predictors of elevated depressive symptoms in the IMID cohort.

Factor	Odds ratio	95% CI	*p*-Value
Sex			
Male	-	-	-
Female	0.79	0.47–1.33	0.34
Age	0.99	0.97–1.00	0.081
Income			
Declined	-	-	-
⩾50,000	1.00	0.47–2.22	0.9
<50,000	2.23	1.03–5.03	0.05
Education			
⩾High school	-	-	-
<High school	1.44	0.92–2.24	0.11
Body Mass Index			
Underweight-Normal	-	-	-
Overweight	1.44	0.86–2.43	0.13
Obese	1.47	0.89–2.46	0.14
Smoking status			
None-moderate	-	-	-
Ever smoker	1.64	1.06–2.56	0.03*
IMID type			
RA	-	-	-
MS	1.08	0.62–1.89	0.91
IBD	0.69	0.38–1.27	0.17
IMID illness duration	1.00	0.98–1.02	0.82
HADS-A Score	1.47	1.38–1.57	<0.001*

Body mass index (data missing for eight participants). Elevated depressive symptoms defined as: HADS-D ⩾ 7 (any IMID) or PHQ-9 ⩾10 (RA), PHQ-9 ⩾11 (IBD), and PHQ-9⩾12 (MS).

We added an interaction term for sex and each factor listed to the adjusted model for the analyses, age ß = 0.02, p = 0.32, BMI-Overweight ß = −0.37, p = 0.62; BMI-Obese ß = 0.45, p = 0.58; IMID-MS ß = 0.91, p = 0.18; IMID-IBD ß = −0.01, p = 0.99; IMID disease duration ß = −0.02, p = 0.47; Education-less than High School ß = 0.80, p = 0.18; Income-<$50,000 ß = −0.18, p = 0.78; Income-⩾$50,000 ß = −0.31, p = 0.79; Smoking ß = −0.65, p = 0.28; HADS-A score ß = −0.07, p = 0.43.

RA: rheumatoid arthritis; MS: multiple sclerosis; IBD: inflammatory bowel disease; PHQ-9: Patient Health Questionnaire; HADS-A: Hospital Anxiety and Depression Scale.

* Significance level *p* < 0.05.

**Figure 1.**
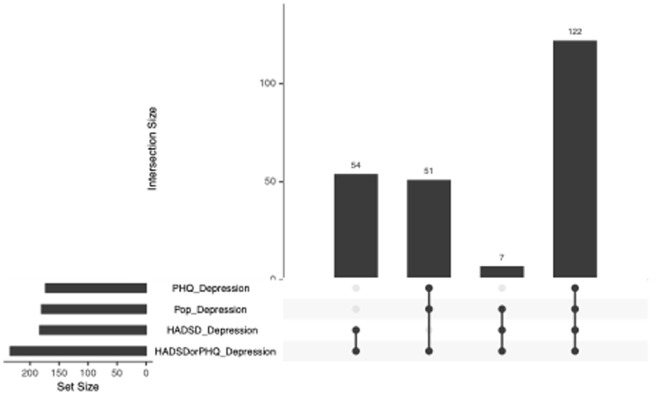
Sensitivity analysis of the various criteria for defining elevated depressive symptoms in the IMID cohort. *Note*. PHQ: Patient Health Questionnaire; HADS-D: Hospital Anxiety and Depression Scale; pop: population. PHQ_Depression: total score on PHQ-9 ⩾ 12/11/10 (MS, IBD, RA); Pop_Depression: total score on PHQ-9 ⩾ 10 or HADS-D ⩾ 11; HADSD_Depression: total score on HADS-D ⩾ 7; HADSDorPHQ_Depression: total score on PHQ-9 ⩾ 12 (MS)/11 (IBD)/10 (RA) or HADS-D of ⩾7.

Both univariable and multivariable regression models showed that experiencing anxiety was statistically significant in predicting elevated depressive symptoms for both males and females (*p* < 0.001). Specifically, for every one unit increase on the HADS-A total score, the odds of experiencing comorbid elevated depressive symptoms in those with an IMID increased by almost 50%. These results remained significant in the sensitivity analysis (Figure 1).

### Sensitivity analyses

In the sensitivity analysis, similar rates of elevated depressive symptoms were identified using other case definitions (range by definition: 27%–38%, Figure 1). In the univariable regression models, using only the HADS-D scale to identify elevated depressive symptoms showed similar findings to the primary analysis (Tables 5 in Appendix 1), obesity and smoking were no longer significantly associated with depressive symptoms using only the PHQ-9 definition (Table 6 in Appendix 1), and no changes were seen with the general population cut-offs for either the PHQ-9 or HADS-D (Table 7 in Appendix 1). In the multivariable regression models, using only the HADS-D scale to identify elevated depressive symptoms showed similar findings to the primary analysis (Tables 8 in Appendix 1), annual income (*p* = 0.03) and being overweight (*p* = 0.02) were significant using only the PHQ-9 while smoking became non-significant (Table 9 in Appendix 1), and the general population cut-offs for both the PHQ-9 and HADS-D showed that income became a significant predictor of elevated depressive symptoms (*p* = 0.02, Table 10 in Appendix 1).

## Discussion

This study aimed to determine which clinical and sociodemographic factors were associated with elevated depressive symptoms in IMIDs, and whether these associations differed by sex, and lastly, if there were any sex differences in the endorsement of individual depressive symptoms. While the high prevalence of elevated depressive symptoms in IMID populations is well-established (Bhandari et al., 2017; Choi et al., 2019; Kowalec et al., 2022; Marrie et al., 2019), our findings add nuance by exploring sex differences across three IMIDs. Based on studies from the general population, we hypothesized females would show higher rates of depressive symptoms than males. Contrary to our hypothesis, females in the IMID cohort were not at a statistically significant increased odds for elevated depressive symptoms compared to males. Elevated anxiety symptoms and smoking were associated with elevated depressive symptoms in all participants, and this did not differ by sex. Males were more likely to endorse the HADS-D item pertaining to cheerfulness than females.

Females with an IMID reported higher elevated depressive symptoms compared to males; however, regression analyses did not find that this was significantly different. One possible explanation is while females are generally more likely to report depressive symptoms, this difference may be attenuated in the context of chronic illness, where symptom burden and psychosocial stressors could affect typical sex-related patterns (Kuehner, 2017; Patten et al., 2018). Additionally, underrepresentation of males in the sample may have limited statistical power to detect differences. Prior studies examining depressive symptoms in IMID populations (specifically those with MS) with a consideration for sex differences have yielded mixed results (Mayo et al., 2021; Solaro et al., 2016; Théaudin et al., 2016). Each IMID included in our study (MS, RA, IBD) has unique pathophysiological mechanisms, symptom trajectories, and associated psychosocial challenges. Sex differences in depressive symptoms may be influenced by disease-specific factors such as the age of onset, hormonal influences, or the social impact of symptoms (i.e. mobility impairment in MS vs gastrointestinal symptoms in IBD; Mikocka-Walus et al., 2016; Skokou et al., 2012; van Vollenhoven, 2009). By aggregating these distinct conditions into a single group, we may have reduced our ability to detect sex differences that are specific to a particular IMID, as strong effects in one condition could be counterbalanced by weaker or opposite effects in another. This highlights the importance of conducting disease-specific analyses to more precisely characterize sex differences in depressive symptomatology within each IMID.

In IMID populations, little is known about whether males and females have similar types of symptoms when experiencing elevated depressive symptoms. Our results showed that females were less likely to report being cheerful compared to males based on the HADS-D question of “I feel cheerful.” This finding should be interpreted cautiously, however, given the large number of scale items tested, and should be replicated in larger sample sizes. This finding may reflect a higher prevalence of anhedonia among females with IMIDs, consistent with literature suggesting that women with depression often experience reduced capacity for enjoyment (Nolen-Hoeksema, 2001; Whittle et al., 2011). This could have implications for treatments, including the utility of interventions focused on behavioral activation or positive affect enhancement in female patients.

In the multivariable analysis, anxiety and smoking were significantly associated with increased odds of depressive symptoms. Smoking is a risk factor for RA and IBD, and it appears to impact depressive symptoms, possibly through inflammation, which negatively influences the course of illness (Monteleone et al., 2023). Anxiety disorders are 1.3x more likely to occur in IMID populations than within the general population (Joyees et al., 2023; Marrie et al., 2017). The comorbidity of depressive and anxiety symptoms is well-established in IMID populations.

Overall, these results emphasize the need for clinicians to consider socioeconomic determinants and the influence that lifestyle factors have on mental health. Therefore, it is recommended that a collaborative care approach encompassing disease-specific interventions and attention to these underlying risk determinants be implemented for comprehensive mental health management in individuals with IMIDs.

### Strengths and limitations

This study had several strengths. We included three IMID types and used two different validated measures of depressive symptoms. As well, this study was novel in probing sex differences on individual items of the implemented depressive symptom scales. However, males were underrepresented and may have affected generalizability of the findings and attenuated any sex-related differences. Increasing sample size and including more males would improve statistical power to detect differences in each group. Although the self-report measures used (PHQ-9 and HADS-D) are validated screening measures for depression and identify depressive symptoms, their use also introduces the possibility of social desirability bias, which could affect the accuracy of results. As well, the cross-sectional design of the study restricts any casual conclusions.

### Future implications and conclusions

Although sex was not associated with elevated depressive symptoms in those with an IMID, smoking and elevated anxiety symptoms were. These observations may help identify individuals at increased risk of elevated depressive symptoms, thereby supporting early intervention. These findings highlight the importance of routine screening for depression and anxiety in individuals with IMIDs, particularly among those with modifiable risk factors such as smoking. These observations may also help inform future clinical guidelines aimed at integrating mental health assessments into chronic illness care. Future studies should further explore tailored, sex-informed mental health interventions across IMID subtypes.

## Appendix 1

**Table 5.** Sensitivity analyses using univariable logistic regression to identify factors associated with elevated depressive symptoms in the IMID cohort using only the HADS-D ⩾ 7 to define elevated depressive symptoms.

	Odds ratio	95% CI	*p*-Value
*Sex*			
Male	-	-	-
Female	1.08	0.73–1.63	0.70
Age	1.00	0.98–1.01	0.56
*Income*			
Declined	-	-	-
⩾50,000	0.84	0.46–1.61	0.59
<50,000	1.34	0.71–2.61	0.38
*Education*			
⩾High school	-	-	-
<High school	1.66	1.16–2.37	0.01*
*BMI*			
Underweight-Normal	-	-	-
Overweight	1.40	0.91–2.16	0.13
Obese	1.83	1.19–2.82	0.01*
*Smoking status*			
None-Moderate	-	-	-
Ever smoker	2.04	1.42–2.96	<0.001*
*IMID type*			
RA	-	-	-
MS	0.95	0.62–1.47	0.82
IBD	0.68	0.43–1.07	0.09
IMID illness duration	1.00	0.99–1.02	0.81
HADS-A score	1.40	1.32–1.48	<0.001*

IMID cohort *N* = 652, elevated depressive symptoms group *n* = 183, non-elevated depressive symptoms group *n* = 469.

RA: rheumatoid arthritis; MS: multiple sclerosis; IBD: inflammatory bowel disease; HADS-A: Hospital Anxiety and Depression Scale.

* Significance level *p* < 0.05.

**Table 6.** Sensitivity analysis using univariable logistic regression models of predictors of elevated depressive symptoms in the IMID cohort using only the PHQ-9 to define elevated depressive symptoms.

	Odds ratio	95% CI	*p*-Value
*Sex*			
Male	-	-	-
Female	1.39	0.92–2.15	0.13
Age	0.98	0.96–0.99	<0.001*
*Income*			
Declined	-	-	-
⩾50,000	0.69	0.37–1.35	0.26
<50,000	1.80	0.95–3.55	0.08
*Education*			
⩾High school	-	-	-
<High school	1.59	1.11–2.28	0.01*
*BMI*			
Underweight-Normal	-	-	-
Overweight	1.50	0.98–2.30	0.07
Obese	1.41	0.91–2.20	0.12
*Smoking status*			
None-moderate	-	-	-
Ever smoker	1.42	0.99–2.05	0.06
*IMID type*			
RA	-	-	-
MS	1.01	0.65–1.59	0.96
IBD	0.79	0.50–1.26	0.32
IMID illness duration	0.99	0.98–1.01	0.21
HADS-A score	1.42	1.34–1.51	<0.001*

IMID cohort *N* = 652, elevated depressive symptoms group *n* = 173; non-elevated depressive symptoms group *n* = 479.

RA: rheumatoid arthritis; MS: multiple sclerosis; IBD: inflammatory bowel disease; HADS-A: Hospital Anxiety and Depression Scale; Disease-specific cutoffs: PHQ-9 ⩾10 (RA), PHQ-9 ⩾11 (IBD), PHQ-9⩾12 (MS).

* Significance level *p* < 0.05.

**Table 7.** Sensitivity analysis using univariable logistic regression models of predictors of elevated depressive symptoms in the IMID cohort using population cut-offs for both the HADS-D and the PHQ-9 to define elevated depressive symptoms.

	Odds ratio	95% CI	*p*-Value
*Sex*			
Male	-	-	-
Female	1.30	0.87–1.99	0.21
Age	0.98	0.96–0.99	<0.001*
*Income*			
Declined	-	-	-
⩾50,000	0.75	0.40–1.46	0.38
<50,000	1.87	1.00–3.69	0.059
*Education*			
⩾ High school	-	-	-
< High school	1.78	1.25–2.55	0.002*
*BMI*			
Underweight-Normal	-	-	-
Overweight	1.50	0.98–2.30	0.07
Obese	1.63	1.06–2.53	0.03*
*Smoking status*			
None-Moderate	-	-	-
Ever smoker	1.55	1.08–2.23	0.02*
*IMID type*			
RA	-	-	-
MS	1.02	0.66–1.60	0.91
IBD	0.76	0.48–1.20	0.24
IMID illness duration	0.99	0.98–1.01	0.25
HADS-A score	1.41	1.33–1.50	<0.001*

IMID cohort *N* = 652, elevated depressive symptoms group *n* = 180; non-elevated depressive symptoms group *n* = 472.

RA: rheumatoid arthritis; MS: multiple sclerosis; IBD: inflammatory bowel disease; HADS-A: Hospital Anxiety and Depression Scale; Population cut-offs: PHQ-9 score of ⩾10, HADS-D score of ⩾11.

* Significance level *p* < 0.05.

**Table 8.** Sensitivity analysis using multivariable logistic regression of predictors of elevated depressive symptoms in the IMID cohort using only the HADS-D ⩾ 7 to define elevated depressive symptoms.

	Odds ratio	95% CI	*p*-Value
*Sex*			
Male	-	-	-
Female	0.62	0.36–1.07	0.08
Age	1.00	0.98–1.02	0.99
*Income*			
Declined	-	-	-
⩾50,000	1.09	0.50–2.49	0.84
<50,000	1.61	0.73–3.74	0.25
*Education*			
⩾High school	-	-	-
<High school	1.42	0.90–2.22	0.13
*BMI*			
Underweight-Normal	-	-	-
Overweight	1.29	0.76–2.22	0.35
Obese	1.63	0.97–2.75	0.07
*Smoking status*			
None-Moderate	-	-	-
Ever smoker	1.68	1.07–2.66	0.03*
*IMID type*			
RA	-	-	-
MS	1.16	0.66–2.04	0.62
IBD	0.65	0.35–1.21	0.17
IMID illness duration	1.00	0.98–1.02	0.74
HADS-A score	1.41	1.33–1.51	<0.001*

IMID cohort *N* = 652, elevated depressive symptoms group *n* = 183; non-elevated depressive symptoms group *n* = 469.

RA: rheumatoid arthritis; MS: multiple sclerosis; IBD: inflammatory bowel disease; HADS-A: Hospital Anxiety and Depression Scale.

* Significance level *p* < 0.05.

**Table 9.** Sensitivity analysis using multivariable logistic regression of predictors of elevated depressive symptoms in the IMID cohort using disease-specific cut-offs for only the PHQ-9 to define elevated depressive symptoms.

	Odds ratio	95% CI	*p-*Value
*Sex*			
Male	-	-	-
Female	1.02	0.58–1.83	0.94
Age	0.97	0.95–0.99	0.01*
*Income*			
Declined	-	-	-
⩾50,000	0.72	0.32–1.69	0.44
<50,000	2.60	1.15–6.19	0.03*
*Education*			
⩾High school	-	-	-
<High school	1.26	0.78–2.02	0.34
*BMI*			
Underweight-Normal	-	-	-
Overweight	1.90	1.10–3.32	0.02*
Obese	1.42	0.82–2.47	0.21
*Smoking status*			
None-moderate	-	-	-
Ever smoker	1.26	0.79–2.02	0.34
*IMID type*			
RA	-	-	-
MS	1.15	0.64–2.09	0.64
IBD	0.75	0.39–1.47	0.40
IMID illness duration	1.00	0.98–1.02	0.96
HADS-A score	1.42	1.33–1.52	<0.001*

IMID cohort *N* = 652, elevated depressive symptoms group *n* = 173; non-elevated depressive symptoms group *n* = 479.

RA: rheumatoid arthritis; MS: multiple sclerosis; IBD: inflammatory bowel disease; HADS-A = Hospital Anxiety and Depression Scale; Disease-specific cutoffs: PHQ-9 ⩾10 (RA), PHQ-9 ⩾11 (IBD), PHQ-9⩾12 (MS).

* Significance level *p* < 0.05.

**Table 10.** Sensitivity analysis using multivariable logistic regression of predictors of elevated depressive symptoms in the IMID cohort using population cut-offs for HADS-D and the PHQ-9 to define elevated depressive symptoms.

	Odds ratio	95% CI	*p*-Value
*Sex*			
Male	-	-	-
Female	0.92	0.52–1.64	0.78
Age	0.97	0.95–0.99	0.003*
*Income*			
Declined	-	-	-
⩾50,000	0.83	0.37–1.95	0.65
<50,000	2.76	1.22–6.58	0.02*
*Education*			
⩾High school	-	-	-
<High school	1.49	0.93–2.38	0.10
*BMI*			
Underweight-Normal	-	-	-
Overweight	1.94	1.12–3.39	0.02*
Obese	1.79	1.04–3.12	0.04*
*Smoking status*			
None-moderate	-	-	-
Ever smoker	1.38	0.87–2.22	0.17
*IMID type*			
RA	-	-	-
MS	1.10	0.61–2.00	0.75
IBD	0.67	0.34–1.30	0.23
IMID illness duration	1.00	0.98–1.02	0.89
HADS-A Score	1.42	1.33–1.52	<0.001*

IMID cohort *N* = 652, elevated depressive symptoms group *n* = 180; non-elevated depressive symptoms group *n* = 472.

RA: rheumatoid arthritis; MS: multiple sclerosis; IBD: inflammatory bowel disease; HADS-A: Hospital Anxiety and Depression Scale; Population cutoffs: PHQ-9 score of ⩾10, HADS-D score of ⩾11.

* Significance level *p* < 0.05.
